# Coordinating COVID-19 vaccine deployment through the WHO COVID-19 Partners Platform

**DOI:** 10.2471/BLT.21.285550

**Published:** 2021-03-01

**Authors:** M Anne Yu, Angela K Shen, Michael J Ryan, Linda Lucy Boulanger

**Affiliations:** a Health Emergencies Programme, World Health Organization, Avenue Appia 27, 1211 Geneva, Switzerland.

Severe acute respiratory syndrome coronavirus 2 (SARS-CoV-2), the virus that causes the coronavirus disease 2019 (COVID-19), is having a deep impact across the globe.

As of 4 February 2021, over 103 million confirmed cases and 2.25 million deaths due to COVID-19 had been reported.^1^ The pandemic has also affected economic productivity and contributed to the deepest global recession since the Second World War.^2^ The human and financial toll of the pandemic has highlighted the need for a model to support coordinated international emergency response.^3^

On 30 January 2020, the World Health Organization (WHO) declared a Public Health Emergency of International Concern; in early February 2020, it launched its strategic preparedness and response plan and the accompanying operational guidelines. This plan outlined key public health measures to guide efforts of national and international partners.^4^^,^^5^ WHO, in collaboration with the United Nations (UN) Development Coordination Office, launched the WHO COVID-19 Partners Platform to operationalize the plan.^6^ On this platform, for the first time, governments, UN agencies and partners can plan and coordinate, in real time, for an acute event. This innovative digital ecosystem operationalizes the nine pillars of the plan: country-level coordination, planning and monitoring; risk communication and community engagement; surveillance, rapid-response teams and case investigation; points of entry, international travel and transport; national laboratories; infection prevention and control; case management; operational support and logistics; and maintaining essential health services and systems. 

The platform has facilitated actions within these initial nine strategic pillars and continues to do so.^6^ As a tool for emergency management, the platform supports transparency, collaboration and efficiency for countries, UN agencies, implementing partners and donors in their COVID-19 response. At the core of the platform is a centralized digital environment where countries and partners, in the context of an outbreak, can: (i) develop and share plans; (ii) monitor and review implemented actions; (iii) cost plans, share resource needs, and request critical supplies; and (iv) view and track donor contributions. These four basic functionalities are fundamental to emergency response in every nation, regardless of whether resources come from the international community or are domestically available. 

As of 4 February 2021, the platform has tracked 9.3 billion United States dollars (US$) of requested resources and approximately US$ 8 billion in donor contributions.^6^ A total of 120 countries, areas and territories have integrated the platform into their emergency management cycle (disease prevention and mitigation, preparedness, response and recovery), supporting preparedness and response using a model that optimizes near real-time management of public health emergency needs and demands. The platform allows for rapid response in public health practice, provides a centralized hub to house information for decision-making, and brings stakeholders from across sectors within countries, including donors and key implementing partners, to collaborate using a common planning framework. The platform is accessible to and enabled by countries, regions and partners, and governed by the countries, who determine how to support their own needs.

Coordination is at the centre of the platform and critical to an effective response. In January 2021, a tenth pillar was added to the platform to support national deployment of COVID-19 vaccines when available.

Vaccines are currently being provided to several countries, mostly high-income, for mass vaccination programmes.^7^^,^^8^ Low-income countries expect to obtain their first doses in the coming months through the global COVAX Facility. As a part of the Access to COVID-19 Tools (ACT) Accelerator, the facility aims to accelerate the development and manufacture of COVID-19 vaccines and promote equitable global access to these vaccines.^9^^,^^10^ Setting up vaccination programmes usually takes years. For COVID-19 vaccine deployment, governments have less time to prepare. Preparation includes identifying prioritized groups for vaccination, given the limited vaccine doses; developing service delivery strategies; supporting training and supervision of health-care workers; addressing product-specific cold chain and logistics requirements; and supporting demand generation including advocacy, communication and community engagement about these novel vaccines.^10^ The current situation has forced governments to prepare COVID-19 vaccination programmes within months. Coordinating needs and resources is a complex task, let alone with the additional challenges of the unknowns about candidate vaccine products and the process countries are to use to secure vaccine doses. Clinical trials and regulatory authorizations are taking place in real time, as are efforts designed to provide equitable access to COVID-19 diagnostics, treatments and vaccines through the ACT Accelerator.^11^^,^^12^

With the coordinated sharing of expertise and resources at national, regional and international levels, the novel digital platform is a new way of responding to emergencies: a way that can decrease disparities in readiness among countries that are struggling in their response to mitigate the spread and impact of the virus.
